# Traditional Uses, Bioactive Constituents, Biological Functions, and Safety Properties of *Oviductus ranae* as Functional Foods in China

**DOI:** 10.1155/2019/4739450

**Published:** 2019-06-02

**Authors:** Yang Zhang, Yifei Wang, Mingzhu Li, Shuyue Liu, Jialu Yu, Zhaowei Yan, Hongli Zhou

**Affiliations:** ^1^School of Biology and Food Engineering, Changshu Institute of Technology, Changshu 215500, China; ^2^Department of Pharmacy, The First Affiliated Hospital of Soochow University, Suzhou 215006, China; ^3^Jilin Engineering Research Center for Agricultural Resources and Comprehensive Utilization, Jilin Institute of Chemical Technology, Jilin 132022, China

## Abstract

*Oviductus ranae* is an animal-based traditional Chinese material widely used as tonics in China for hundreds of years. Various bioactive components are present in OR including proteins, amino acids, steroids, fatty acids, phospholipids, nucleosides, vitamins, hydantoins, and mineral elements. These constituents exert a myriad of biological functions such as immunomodulatory, antioxidant, antifatigue, antiaging, estrogen-like, hepatoprotective, hypolipidemic, antiosteoporotic, antidepressant, antitumor, antitussive, expectorant, anti-inflammatory, and antiasthmatic activities. Unlike other traditional Chinese crude drugs recorded in Chinese Pharmacopoeia, OR is seldom prescribed as medicine but often consumed as nutraceuticals to optimize health. In this review, the traditional uses, bioactive constituents, biological functions, and safety properties of OR as functional foods in China were summarized and discussed. It is expected that this review will provide useful information for anyone who is interested in OR.

## 1. Introduction

Natural products (NPs) are substances derived from plants, animals, and microorganisms [1]. NPs play an important role in human disease prevention and treatment, which provide the sources of lead compound discovery for most of the modern medicines and functional foods [2, 3]. Approximately 25% of the drugs approved by the Food and Drug Administration (FDA) and/or European Medicines Agency (EMA) were of plant origin, such as the well-known morphine and paclitaxel [4]. Meanwhile, plant-based nutraceuticals and functional foods have gained an increasing attention due to their better safety profiles and therapeutic potentials, such as polyphenols and terpenoids [3, 5]. Actually, in addition to plant, animals and/or products derived from their organs are essential constituents in the preparation of many traditional medicines and tonics, especially for the prevention and healing of chronic diseases [6, 7], for example, the dried *Agkistrodon Japonicae* has been traditionally consumed in Japan as a tonic for relieving physical fatigue. *Phocae* Testis et Penis is the dried penis and testis of *Phoca vitulina* or *Callorhinus ursinus* and has been traditionally used as a tonic and as an invalid food for a long time [8]. Thus, animal-derived NPs equally deserve more attention, particularly evidence-based traditional ones.

Animal-based crude drugs constitute a significant part of complementary and alternative medicine in China. To date, about 1850 animals were recorded with therapeutic values in China, 93 animal species, and 369 formulae containing animal-based drugs were listed in Chinese Pharmacopoeia 2010 edition [9]. Among them, *Oviductus ranae* (OR), the dried oviduct of mature female *Rana dybowskii*, also known as Hamayou and Hashimayou, is a famous traditional animal-based medicine, which has been used in China for over hundreds of years to treat or prevent various ailments including debilitation, night sweat, neurasthenia, insomnia, and climacteric syndrome [10, 11].

**Figure 1:**
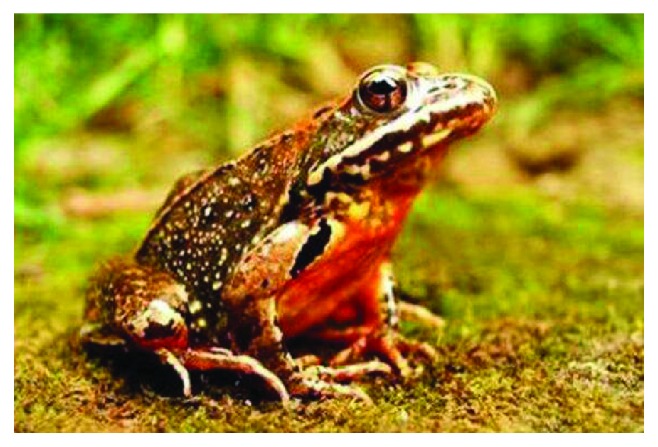
*R. dybowskii*. The figure was obtained from https://image.baidu.com/, the search keyword was “*R. dybowskii*”.

China is home to an enormous diversity of amphibian species with 321 species of frogs and toads [12]. There are about 20 subspecies of Chinese brown frogs distributed in south, northeast, and northwest China, such as *R. chensinensis*, *R. kukunoris*, *R. dybowskii*, *R. zhenhaiensis*, *R. chaochiaoensis*, *R. huanrenensis*, *R. amurensis*, and *R. omeimontis* [13–16]; of which *R. dybowskii*, the Chinese brown frog that lives in northeastern China, enjoys a high reputation due to the fact that its dried oviduct is defined as OR in Chinese Pharmacopoeia [10]. In the past, *R. chensinensis* was confused as the origin of OR. In 1999, Xie et al. restored the species name *R. dybowskii*, which is also called “Hama” and “Hashima,” one kind of small amphibious frogs with the body length of mature male ranging from 52 mm to 64 mm and female ranging from 58 mm to 64 mm (Figure 1) [17, 18]. Natural populations of *R. dybowskii* are special amphibian in northeastern China, mainly distributed in mountain and wet woodlands at low altitudes of 600 m~1300 m. With the aim of protecting natural resources of OR, the wild *R. dybowskii* was listed as one of the national key-protected wild medicinal materials by the Chinese government in 1987. The artificial breeding of wild *R. dybowskii* succeeded and achieved large-scale reproduction in the 1990s. Nowadays, the largest *R. dybowskii* farming area worldwide is in Changbai and Xiao Hinggan mountains in northeastern China [15, 19].

**Figure 2:**
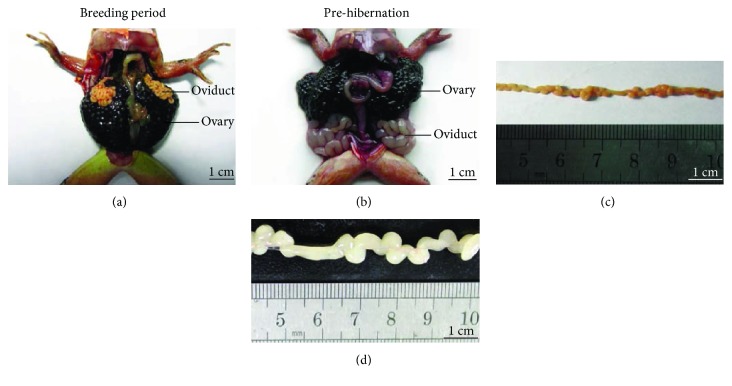
Morphological changes of the oviduct (*Oviductus ranae*) from *R. dybowskii* [20].

**Figure 3:**
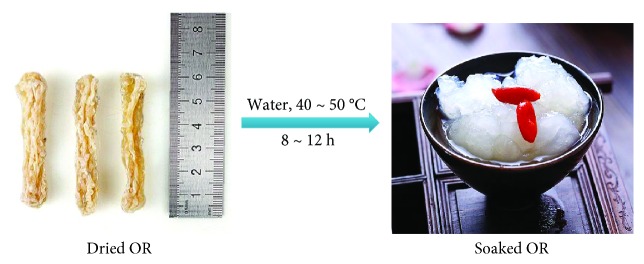
Diagram of the preparation process of edible *Oviductus ranae* from dried product.

The economic value of farmed *R. dybowskii* chiefly depends on the quality of its oviduct (*Oviductus ranae*, OR). *R. dybowskii* is a migrator between mountain and wetland; its hibernation usually starts from October to February, followed by a breeding period spanning from February to June resting with the altitude and latitude. Unexpectedly, the oviduct of *R. dybowskii* usually expands during the period of prehibernation, instead of the breeding period (Figure 2). Meanwhile, levels of some immunomodulatory cytokines and receptor proteins, including interleukin- (IL-) 1*β*, interleukin-1 receptor type 1 (IL1R1), peroxisome proliferator-activated receptor- (PPAR-) *γ*2, leptin, and leptin receptor, are also higher in the oviduct during prehibernation compared with the breeding period [20, 21]. Thus, it is the optimum moment to harvest OR from *R. dybowskii* in autumn. After being washed, fresh OR is dried in shade or using freeze-drying process. The dried OR can be grinded into powder to prepare medicinal prescriptions or can be soaked in warm water and mixed with flavors for oral consumption (Figure 3) [22].

In China, OR has been recorded in Chinese Pharmacopoeia as a traditional Chinese medicinal material since the year 1985; however, it is not usually used as a medicine to treat diseases but often consumed as a tonic to optimize health. Enormous studies have been carried out to investigate the chemical compositions and biological functions of OR, coupled with the research and development of new nutraceutical products based on OR. In this article, we made an overview on the traditional uses, bioactive constituents, biological functions, and safety properties of OR as functional foods in China. It is hoped that this review will provide some basic knowledge and useful information for anyone who is interested in the further investigation and commercial exploitations of OR.

## 2. Traditional Uses of *Oviductus ranae*

*R. dybowskii* was first descripted as “Shanha” in Bencao Tujing in 1061 A.D. and mentioned as “Hashima” in Complete Library in the Four Branches of Literature in 1782 A.D. [23]. In the Qing dynasty, OR was used as a precious tonic and a tribute paid to the royals [19]. In traditional Chinese medicine (TCM), OR is known to possess a sweet, salty, and neutral taste and passes through the kidney and lung channels. TCM holds the opinion that OR can nourish the kidney and strengthen the essence, moisten the lung to nourish Yin. Based on its channel tropism, traditionally, OR is mainly consumed to relieve the symptoms associated with debilitation, phthisis, cough, hematemesis, and night sweat. It is also combined with other Chinese medicinal materials to facilitate and improve the synergistic actions of each crude drug, e.g., with *Tremella fuciformis* to treat phthisis, with Indian swiftlet saliva to cure neurasthenia, and with *Codonopsis pilosula*, donkey-hide gelatin, *Atractylodes macrocephala*, and *Astragalus mongholicus* to relieve severe night sweat [24]. OR has been listed in Chinese Pharmacopoeia since 1985, and it is still recorded as a traditional Chinese medicinal material in the current edition of Chinese Pharmacopoeia [10, 25].

## 3. Bioactive Constituents in *Oviductus ranae*

So far, the bioactive compounds in OR have not been fully elucidated. With the advances of modern analytical techniques, various classes of bioactive constituents including mineral elements, amino acids, proteins, steroids, fatty acids, phospholipids, nucleosides, vitamins, and hydantoins have been isolated and identified in previous studies. All the chemical ingredients present in OR are shown in Table 1.

### 3.1. Mineral Elements

Approximately 21 mineral elements were found in OR by atomic absorption spectrometry, inductively coupled plasma atomic emission spectrometry (ICP-AES), and ICP mass spectrometry (ICP-MS) (Table 1). Contents of Ca, K, Na, Mg, and Fe were higher than other elements [53].

Shen et al. determined the content of Ca, Mg, Mn, Fe, Cu, Zn, Cr, and Pb in OR, respectively, and found that the content of Ca is 4201.37 *μ*g/g with the highest level and Pb is 0.53 *μ*g/g [29], which is within the scope of the maximal residue limit of Pb in food (0.02~2.0 *μ*g/g) [60]. Fan et al. observed that the Zn/Cu ratio in OR is 0.57, which is lower than that in terrestrial angiosperm (11.4), and the Zn/Cd ratio is 25.03, suggesting that long-term intake of OR might exert beneficial effects on cardiovascular health [61–63].

### 3.2. Amino Acids and Proteins

In OR, amino acids and proteins are the main components associated with tonic effects; in most cases, the contents of proteins are higher than 50% [19, 31, 64]. There are totally 18 amino acids in OR, eight of them are essential amino acids, which account for 45.5% (Table 1). Among them, the total contents of hydrophobic amino acids Gly, Ala, Val, Leu, Ile, Pro, and Phe are 157.1 mg/g with Pro in the highest level of 32.3 mg/g. The basic amino acids Lys, Arg, and His have a total content of 43.5 mg/g with Lys in the highest content of 21.1 mg/g, and the acidic amino acids Glu and Asp have a total content of 81.1 mg/g with Asp in the highest level of 41.8 mg/g. The total contents of other amino acids including Trp, Met, Thr, Ser, Cys, and Tyr are 143.1 mg/g with Thr the highest content of 57.0 mg/g [54]. It has also been found that the water-soluble proteins in OR (WSPOR) take up 13.3%, which consist of 15 kinds of amino acids [61].

A number of studies have been conducted to research WSPOR and noted that most of them belonged to glycoproteins [65]. Wang et al. prepared WSPOR by water extraction and ultracentrifugation. Four bands were detected by using sodium dodecyl sulfate polyacrylamide gel electrophoresis (SDS-PAGE), and the molecular weights were found to be 170, 157, 128, and 49 kDa, respectively [33]. Zheng et al. precipitated the crude glycoproteins from oviductal flushing fluid by saturated ammonium sulfate. After being fractionated by column chromatographies on diethylaminoethyl cellulose (DEAE-C) and Sephadex G-100, a single peak (OGP-I) with a molecular weight of 116 kDa was obtained. OGP-I can immunize New Zealand albino rabbit to generate polyclonal antibodies with an enzyme-linked immunosorbent assay (ELISA) titer of 1 : 64000 [34, 35]. Li et al. extracted WSPOR by repeated thawing and freezing. After purification, a single protein (ROGP-III) with a molecular weight of 66 kDa was obtained. The purity of ROGP-III was 97.2%, and the content of polysaccharides was 17% [36]. Moreover, a specific protein with a molecular weight of 20.2 kDa (signal peptidase I) was isolated and identified from OR by Lin et al. using gel electrophoresis coupled with nanoliquid chromatography/mass spectrometry, which has the potential to be developed as a marker of distinguishing OR from its counterfeits [38].

### 3.3. Steroids

Steroids are a kind of natural products widely distributed in animals and plants. They are characterized by a special cyclopentano-perhydrophenanthrene skeleton and comprise a variety of chemical structures and include some vital compounds necessary for human life, such as glucocorticoids, sex hormones, bile acids, cholesterol, and vitamin D. Steroids possess important biological functions including various hormonal activities, lipid digestion and absorption, and heart failure treatment, as well as cell membrane stability, growth, and proliferation [66].

Several steroidal compounds including cholesterol, cholesteryl palmitate, estradiol, and progesterone have been isolated or detected in OR (Table 1), which give OR the properties of nourishing Yin and strengthening Yang [45, 67]. Zhao et al. isolated three steroidal compounds, namely, cholesterol, cholest-4-ene-3-one, and cholestan-3,6-dione, from the petroleum ether extract of OR, and the latter two compounds were firstly isolated from OR [42]. Wang et al. isolated two new steroids from OR using column chromatography on Sephadex LH-20 and octadecylsilyl, coupled with pre-high-performance liquid, and elucidated their chemical structures to be cholest-5-ene-3*β*, 7*β*-diol, and 3*β*-hydroxyl-cholest-5-ene-7-one [11]. Other steroids identified from OR include 7-dehydrocholesterol, cholesteryl palmitate, and stigmasterol [44]. In addition, sex hormones, such as estradiol, progesterone, testosterone, androsterone acetate, and medrogestone, were also found in OR with estradiol having the highest level of 0.87% [46].

### 3.4. Fatty Acids and Phospholipids

Some studies have focused on the fatty acids in OR, especially for unsaturated ones. 24 kinds of fatty acids with a wide range of carbon chain lengths (C_7_~C_22_) have been detected in OR using gas chromatography (GC) or GC mass spectrometer (GC-MS) (Table 1). Liang et al. compared the fatty acid composition in the oviduct of *R. dybowskii* (OR), *R. nigromaculata*, and *Bufo gargarizans* and found that the unsaturated fatty acid content in OR is 64.28%, more than that in the oviduct of *Bufo gargarizans* (43.01%), little less than that in the oviduct of *R. nigromaculata* (65.74 %), and OR contains the highest content of essential fatty acids compared with the oviducts of *R. nigromaculata* and *Bufo gargarizans*. Hou et al. observed that the contents of monounsaturated fatty acids in OR are 38.18% with oleic acid in the highest level of 32.08%, and the polyunsaturated fatty acids are 27.95% with linoleic acid in the highest level of 22.1%, suggesting that consumption of OR might exert cardioprotective effects [68–70].

Three kinds of phospholipids including sphingomyelin, cephalin, and phosphatidylcholine were found in OR, but their total content is much lower than that in egg oil of *R. dybowskii* [52].

### 3.5. Vitamins

Several vitamins, such as vitamin A, D, E, K, B_1_, B_2_, and *β*-carotene, were detected in OR with vitamin E in the highest level of 100.5 mg/g, which may be one of the important functional compositions relating to insomnia and climacteric syndrome treatment in OR [54, 71].

### 3.6. Hydantoins

In traditional Chinese medicinal materials, many constituents naturally coexist with each other; in order to facilitate quality evaluation, some constituents with high content or exclusive properties are usually selected as indicative constituents, and 1-methylhydantoin (1-MID) is chosen as the indicative constituent in OR [25]. Gao et al. compared the contents of 1-MID in different parts of *R. dybowskii* and found that the content of 1-MID in OR is 4.7 *μ*g/g, less than those in other parts including the head, egg oil, body, and limbs [72].

### 3.7. Others

In addition to the above-mentioned steroid hormones, some other hormones including human chorionic gonadotropin, thyroxin, triiodothyronine, and parathyroid hormone were also found in OR [53, 54]. Moreover, crude polysaccharides from OR were prepared by enzyme-assisted extraction, and it was noted that the polysaccharide yield of neutral proteinase-assisted extraction (10.4%) is higher than that of trypsin-assisted extraction (7.2%) [58, 59]. The nucleosides, such as inosine, were also observed to be present in the methanolic extract of OR with content of 23.31 *μ*g/g [55].

## 4. Biological Functions of *Oviductus ranae*

Recently, an increasing number of articles have been reported on the biological functions of OR and its active principles on immunomodulatory, antioxidant, antifatigue, antiaging, estrogen-like, hepatoprotective, hypolipidemic, antiosteoporotic, antidepressant, antitumor, antitussive, expectorant, anti-inflammatory, and antiasthmatic activities. These findings indicated that OR has immense potential for further research and development. A summary on the modern bioactive studies of OR is listed in Table 2.

### 4.1. Immunomodulatory Activities

The immune system is extremely important for the defense of pathogens that threaten lives, and it is thought that about 98% of animal species have acquired innate immunity [128]. Oral administration of OR and its active principles including peptides, proteins, and protein hydrolysates can regulate the immune functions and phagocytic capacities of macrophages. Wang et al. reported that OR in doses of 0.67 and 1.33 g/kg BW can enhance the hemolytic activity, antibody-producing cells, lymphocyte proliferation, delayed-type hypersensitivity response, NK cell activity, and macrophage phagocytosis but shows no effect on immune organ indexes in normal mice over a period of 30 days [73]. However, in mice of cyclophosphamide-induced immunosuppression, oral administration of OR in dose of 0.1 g/k BW for 8 days significantly increased the immune organ indexes and macrophage phagocytosis [129]. These results indicated that OR exerts immunomodulatory effects both in normal and immunosuppressional model mice, repairs the atrophied immune organs caused by immunodeficiency, and has no effect on normal immune organs. In addition, protein from OR and its hydrolysates prepared by different hydrolases can increase spleen lymphocyte proliferation, macrophage phagocytosis, and productions of IL-1*β*, IL-6, TNF-*α*, NF-*κ*B, and NO. The underlying mechanism may be involved in upregulating the mRNA and protein expression of iNOS and stimulating macrophage activities via activating the NF-*κ*B pathway [77].

Immune-enhancement effect is one of the most comment functions applied in OR-related functional foods in China. For example, oral administration of Hamayou soft capsule in a dose of 4 g/day increases CD_3_^+^ and CD_4_^+^ and the ratio of CD_4_^+^/CD_8_^+^ in young male athletes for 28 days [80]. Compound Renshen-Hamayou capsule enhances delayed-type hypersensitivity response and spleen lymphocyte proliferation in mice in a dose of 1.5 g/kg BW [79].

In summary, besides OR, its active principles, especially protein hydrolysates, equally exert considerable effect on the immune system. Further studies regarding the research and development of novel immunomodulators derived from OR are necessary.

### 4.2. Antioxidant Activities

Many reports suggested that OR and its principles have antioxidant activities both *in vitro* and *in vivo*. Oral administration of OR in doses of 0.5 and 2.5 g/kg BW for 28 days significantly increased SOD activity and decreased MDA content in mice exposed to repeated cold stress [81]. In aging rats, compound Renshen-Hamayou capsule exhibited antioxidant effects through increasing GSH-Px activity and decreasing MDA level [88], while in mice of X-ray-induced oxidative stress, intragastric administration of compound Linwayou granule in doses of 100 and 200 mg/kg BW for 7 days can also remarkably elevate SOD and GSH-Px activities [89]. Ling et al. explored the molecular mechanisms of the antioxidant activities of OR and observed that OR-containing serum elicits protective effects on oxidative stress-induced apoptosis in rat ovarian granulosa cells through decreasing apoptosis via reducing ROS production and improving mitochondrial membrane potential via downregulating p53, Bax, caspase-3, and caspase-9 and upregulating Bcl-2, as well as weakening the phosphorylation of JNK and p38 MAPK, and enhancing the phosphorylation of ERK1/2 [83]. At the gene level, oral administration of OR can upregulate the expression of antioxidant-related gene Cu/Zn-SOD both in young and in aging mice in different stages. The expression of Cu/Zn-SOD gene reached the highest level of 2.589 ± 0.182 in a dose of 0.45 g/kg BW in young mice on day 15, and in aging mice, the highest level of gene expression (2.923 ± 0.41) was presented in a dose of 1.35 g/kg BW on day 30 [82], implying that the upregulation of Cu/Zn-SOD gene needs higher dose of OR and more time in aging mice than those in young mice due to the fact that the declined SOD activities in aging mice could induce the reduction of SOD-related gene expression [130].

In 2014, Zhang et al. screened the antioxidant components of OR and found that water-soluble proteins from OR (WPOR) exert stronger antioxidant capacities compared with OR and water-insoluble constituents, the free radical-scavenging rates towards hydroxyl, DPPH, and superoxide anion in a concentration of 10 mg/mL were 45%, 37%, and 71%, respectively; they also noted that the WPOR hydrolysates prepared by neutral protease possess higher activities than those prepared by papain and by alkali protease, the free radical-scavenging rates towards hydroxyl, DPPH, and superoxide anion in a concentration of 10 mg/mL were elevated to 60%, 45%, and 82%, respectively [87]. In 2016, Liu et al. further strengthened the evidence that WPOR and salt-soluble proteins from OR elicit higher free radical-scavenging capacities than those of gliadin and alkali-soluble proteins from OR [85]. In addition, Liu et al. used papain and neutral protease to hydrolyze WPOR; after centrifugation, dialysis, and fractionation, two peptides with molecular weights of 1~5 kDa (BPOR1) and 5~8 kDa (BPOR2) were obtained, respectively. Further evaluation of antioxidant capacity verified that BPOR1 with a smaller molecular weight possesses higher activity than BPOR2 [86]. These results indicated that proteins are the main antioxidant constituents present in OR; preparation method, water solubility, and molecular weight distribution may significantly affect the antioxidant activities of WPOR.

**Figure 4:**
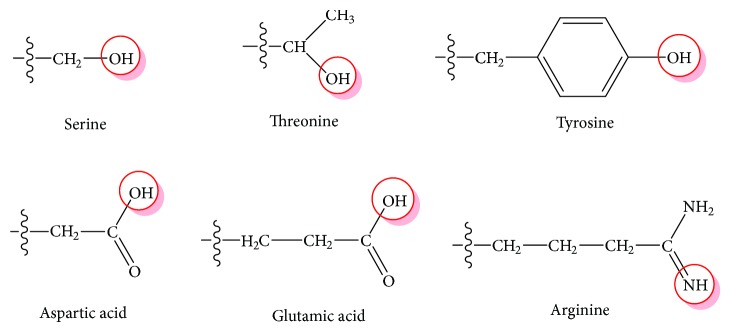
Hydrogen-donor side chains of amino acids present in WPOR [84].

In a previous work [84], we investigated the preparation, amino acid composition, and *in vitro* and *in vivo* antioxidant activities of WPOR and found that WPOR exert weaker *in vitro* radical-scavenging capacities to hydroxyl, superoxide anion, and DPPH as well as ferric-reducing power compared with vitamin C (VC) but show stronger antioxidant effects in an ethanol-induced oxidative stress mouse model. These results were in accordance with the general findings that proteins and polypeptides usually exhibit lower *in vitro* free radical-scavenging capacities than their smaller peptides and amino acids, due to the fact that substances with smaller molecular weight may be prone to interact with free radicals more effectively [131]. We then linked the amino acid composition of WPOR to their antioxidant activities and noted that six amino acids with hydrogen-donor side chains are present in WPOR (Figure 4), which may provide active hydrogens to destroy the *in vitro* free radicals; eight antioxidant amino acids including glutamic acid, aspartic acid, threonine, tyrosine, methionine, histidine, phenylalanine, and lysine that accounted for 52% are present in WPOR, which may play great significance in the *in vivo* antioxidant effects of WPOR. In addition to amino acids, small peptides are also the digestive products of proteins in the body; in the near future, contributions of small peptides absorbed in the intestinal tract to the antioxidant effects of WPOR will need further study to be explored.

### 4.3. Antifatigue Activities

Liu et al. treated kidney Yang deficiency mice induced by hydrocortisone with OR in a dose of 680 mg/kg BW, petroleum ether extract from OR (PEEOR) in doses of 68 and 136 mg/kg BW, and methanol extract from OR (MEOR) in doses of 85 and 170 mg/kg BW for 7 days, respectively. Results showed that OR and PEEOR in a dose of 135 mg/kg BW obviously increased body weight and temperature and the endurance time of rotarod test and FST compared with the model group, but the effects of MEOR were not significant [91], suggesting that the hydrophobic components in OR, especially sex steroids, may be the main constituents that can relieve the symptom of kidney Yang deficiency owing to the fact that sex steroids enhance immunoglobulin synthesis, leading to the improvement of endurance and immune system [132, 133]. In high-intensity exercise training rats, oral administration of OR in doses of 0.5, 1.0, and 3.0 g/kg BW for 6 weeks markedly increased the endurance time of FST and body weight, testosterone, hemoglobin, and hepatic and muscle glycogen and decreased BUN [92]. In normal mice, intragastric administration of OR in doses of 0.25, 0.5, and 1.0 g/kg BW for 30 days can prolong the endurance time of FST, increase liver glycogen, and decrease BLA but have no effects on body weight and BUN. These results implied that OR exerts antifatigue effects both in normal mice and in high-intensity exercise training rats; it can raise the weight loss induced by exercise training and reduce the elevated BUN but has no effects on normal mice. Furthermore, an increasing number of papers have reported that water-soluble proteins, protein hydrolysates, peptides, and water-insoluble components from OR as well as some functional products containing OR equally possess antifatigue effects *in vivo* (Table 3).

Based on the above results, it can be seen that the hydrophobic components and protein-based principles in OR may be the major antifatigue ingredients, but further studies are needed to confirm the assumption and fully elucidate the exact mechanisms.

### 4.4. Antiaging Activities

In 1998, Liu et al. first proved the antiaging effects of OR in *Drosophila melanogaster* in doses of 0.34 and 0.68 mg/g culture medium for an observation period of 40 days. Results showed that OR significantly extends the life-span, enhances the resistance to cold, and increases lipofuscin in *Drosophila melanogaster* [100]. In *D*-galactose-induced aging mice, oral administration of OR in doses of 0.1, 0.2, and 0.4 g/kg BW for 43 days elicited antiaging effects through decreasing MDA and XOD as well as increasing MPO and NO [101]. Its mechanisms in the molecular level may be involved in upregulating the expressions of cyclin D1, CDK6, and p15 proteins as well as downregulating the expressions of p16 and p21 proteins (Table 3).

Qu et al. compared the antioxidant activities of water-insoluble constituents from OR (WICOR) and their hydrolysates in *D*-galactose-induced aging mice in oral doses of 0.15 and 0.3 g/kg BW for 30 days. Results exhibited that hydrolysates obviously decrease MDA and increase SOD and GSH-Px, but WICOR only reduce MDA and have no effects on SOD and GSH-Px. These results indicated that hydrolysates may be inclined to be absorbed in the gastrointestinal tract due to their better fluidity and smaller active components compared with WICOR [107], which deserve to be further investigated. Moreover, water-soluble proteins from OR also decreased MDA and increased SOD in *D*-galactose-induced aging mice in doses of 0.075, 0.15, and 0.3 g/kg BW for 30 days [106].

In summary, taking together the above-mentioned biological functions, the antiaging activities of OR may relate to its immunomodulatory function, antioxidant capacity, regulations of cyclical regulatory proteins and senescence gene expression, and so on.

### 4.5. Estrogen-Like Activities

Kang et al. [110] studied the estrogen-like effects of OR in female Kunming mice in doses of 0.05 and 0.2 g/kg BW for 37 days. It was found that OR can increase the contents of E2, FSH, T, and P and the thickness of the uterine wall and the number of corpora atretica, indicating that OR exerts estrogen-like activities through elevating the hormone levels of hypothalamic-pituitary-gonadal axis thereby improving the reproductive capacity. However, OR in a dose of 0.2 g/kg BW upregulated the expressions of FSHR gene in the adrenal gland and ovary, implying that consumption of high-dose OR may accelerate the development of premature ovarian failure in normal mice [134]. In a different experiment, serum from OR-treated rats (4.5 g/kg BW, i.g., for 5 days) in concentrations ranging from 15% to 30% significantly promoted proliferation, increased E2, and inhibited the apoptosis of ovarian granulosa cells [109]. As summarized above, OR shows estrogen-like activities both *in vitro* and *in vivo*. Some compound preparations, such as *Rhizoma curcumae*-*Oviductus ranae* [111], also exhibited effects in ovariectomized rats, indicating a therapeutic potential in the relief of climacteric syndrome. In terms of premature ovarian failure induced by high-dose consumption of OR in normal mice, additional studies will be needed to verify whether it could increase the incidence of ovarian cancer in normal individuals for long-term and high-dose consumption of OR [135].

### 4.6. Hepatoprotective Activities

In rats with CCl_4_-induced liver fibrosis, intragastric administration of OR in doses of 1.5 and 3.0 g/kg BW for 8 weeks remarkably reduced ALT, AST, ALP, GGT, Hyp, and MDA and elevated SOD and GSH-Px [112]. These results suggested that the hepatoprotective activities of OR may be related to its antioxidant activity, which was further confirmed by another investigation, where Zhang et al. [114] observed that oral administration of OR in doses of 0.9, 4.5, and 9.0 g/kg BW for 28 days can significantly increase SOD and decrease MDA in the liver in high-intensity exercise training mice. Besides, OR in an oral dose of 3 g/kg BW for 8 weeks relieved ethanol-induced liver fibrosis in rats via inhibiting the activation of hepatic stellate cells [113]. Reported researches on the hepatoprotective effects of OR are still limited, but they may give an evidence to expand the existing functional potential of OR.

### 4.7. Hypolipidemic Activities

OR, water-soluble proteins from OR, and water-insoluble constituents and their hydrolysates produced by alkali protease from OR in doses of 0.05, 0.1, and 0.2 g/kg BW for 30 days were all found to significantly decrease the contents of TG and TC in high-fat-diet-induced hyperlipidemic rats. Meanwhile, hydrolysates of water-insoluble constituents produced by alkali protease showed more hypolipidemic effects than water-insoluble constituents, but both OR and its principles had no effects on high-density lipoprotein cholesterol (HDL-C) [115–118]. These results indicated that different parts of OR exert certain hypolipidemic effects, but the mechanism of action should be further investigated.

### 4.8. Antiosteoporotic Activities

Osteoporosis, a metabolic bone disease, is characterized by skeletal fragility and susceptibility to fracture. It has become a major health problem worldwide due to the high morbidity and health care cost involved [136, 137]. The incidence of osteoporosis is in proportion to age and occurs in both men and women, affecting over 12% of men and 40% of women at some point in their lives [138]. Wang et al. [119] treated ovariectomized rats with 0.05 g/kg BW of OR for 12 weeks. The analysis showed that OR obviously increased calcium, estrogen, osteocalcin, BMD, and bone scan index while decreasing ALP, phosphorus, and BGP compared with the ovariectomized group. On the other hand, OR significantly enhanced the proliferation of osteoblastic cells and the formation of mineralized nodes and reduced the number of TRAP-positive cells as well as led to significant differences in bone histomorphometry including femoral weight and W.th. These results indicated that OR plays an important role in the prevention and/or treatment of osteoporosis induced by ovariectomy in rats. The antiosteoporotic activity of OR is similar to that of conjugated estrogens but without affecting uterus weight in ovariectomized rats. This is of great importance since a high level of estrogens can induce endometrial hyperplasia and increase the risk of breast, ovarian, and endometrial cancers [149]. Combining the above-mentioned results in Section 4.5, consumption of OR may possess more safety properties in menopausal women than in normal individuals, which will require further investigations to be explored. In addition, a clinical trial with 30 cases of postmenopausal osteoporosis reported by Lim and Luderer [130] further confirmed the antiosteoporotic effects of OR, whereby the clinical signs and symptoms of patients were significantly improved compared with pretherapy and the group of Xianling Gubao capsule (a well-known antiosteoporotic traditional Chinese drug, 3.0 g per day, for 6 months) after being given 1.0 g of OR per day over a period of 6 months. OR also obviously ameliorated the bone metabolic markers, such as E2, BALP, IL-6, and BGP, but both OR and Xianling Gubao capsule showed no effect on BMD, which may be caused by the short administration time.

### 4.9. Antidepressant Activities

Depression is a common mental disease estimated to rank first in disability-adjusted life years by the year 2020 [139]. Although there are many options of medication, the response to antidepressants is subject to high variables and delayed onset as well as significant adverse effects [140]. Thus, the search for better antidepressants remains an ongoing concern. Recently, natural products have exhibited unique and promising profiles in the treatment of depression, for example, St. John's wort extract has become one of the most commonly used natural product-based antidepressants worldwide [141]. Several reports suggested that OR extracts have antidepressant effects. Intragastric administration of petroleum ether extract from OR to mice in doses of 100, 300, and 900 mg/kg BW for 8 days significantly decreased the dead time of FST and TST as well as antagonized the akinesia and body temperature fall induced by reserpine. These results may be related to the decrease of serum CORT [121]. In a similar study, You et al. treated depression model rats induced by chronic mild stress with 30, 100, and 300 mg/kg BW of petroleum ether extract from OR for 21 days. Results exhibited that petroleum ether extract from OR remarkably increased body weight, motion distance of OFT, and sucrose preference. Its mechanisms may be involved in the decrease of serum CORT and the upregulation of BDNF protein expression [122].

1-Methylhydantoin (1-MID, Figure 4) is the indicative component of OR recorded in Chinese Pharmacopoeia [25]. It was separated from 95% ethanol extract of OR by Wang et al. [56]. Intragastric administration of 1-MID in doses of 20, 40, and 80 mg/kg BW to chronic forced swim stress-induced depression model rats for 14 days obviously increased the body weight and sucrose preference via decreasing serum CORT and upregulating the expression of BDNF protein [123], suggesting that 1-MID is a promising lead compound of novel antidepressant that originated from OR, and further researches associated with lead optimization as well as structure-activity relationship will be needed to investigate. In terms of petroleum ether extract from OR, previous studies have reported that three compounds including cholesterol, cholestan-3,6-dione, and cholest-4-ene-3-one were separated from it [42], which belong to the steroids that elicit antidepressant-like activities [142–144]; however, additional experiments will still be wanted to verify whether these compounds mainly contribute to the antidepressant effect of petroleum ether extract from OR.

### 4.10. Antitussive, Expectorant, Antiasthmatic, and Anti-Inflammatory Activities

Liu et al. compared the antitussive and expectorant effects of OR with its petroleum ether and methanol extracts. The analysis showed that OR significantly prolongs the incubation period of cough, reduces cough frequency, and increases phenol red expectoration and sputum ejection; its extracts also exert certain antitussive (methanol extract > petroleum ether extract) and expectorant effects (petroleum ether extract > methanol extract) [124]. In a mouse model of ovalbumin-induced allergic asthma, oral administration of OR in doses of 0.05 and 0.5 g/kg BW for 8 weeks remarkably decreased inflammatory cell count as well as contents of IL-4, IL-5, and IFN-*γ* in BALF [125]. These results indicated that OR possesses significant antitussive, expectorant, and antiasthmatic effects, which are consistent with the traditional opinion of moistening the lung to nourish Yin, and its mechanisms may be partially related to the anti-inflammatory activity of OR. Meanwhile, methanol extracts from OR may largely contribute to its antitussive effect and petroleum ether extract to expectorant effect, but the exact contributed bioactive constituents are still unclear.

In addition to antidepressant-like activity, 1-MID also exhibited favorable antiasthmatic and antitussive effects. Intragastric administration of 1-MID in doses of 40 and 80 mg/kg BW to a rat model of ovalbumin-induced allergic asthma for 7 days significantly decreased contents of IL-5 and eotaxin as well as eosinophil count in BALF. In a guinea pig model of acetylcholine-induced bronchial asthma, 1-MID in oral doses of 30 and 60 mg/kg BW for 3 days obviously prolonged the incubation period of asthma. In a mouse model of ammonia-induced cough, oral administration of 1-MID in doses of 50 and 100 mg/kg BW for 3 days significantly extended the incubation period of cough and reduced the frequency of cough, while in a guinea pig model of citric acid-induced cough, intragastric administration of 1-MID in doses of 30 and 60 mg/kg BW also elicited antitussive effect. *In vitro*, 0.5 and 1.0 g/L of 1-MID can relax the contraction of guinea pig tracheal smooth muscle induced by histamine [126]. These results implied that the antiasthmatic effect of 1-MID may be associated with the inhibition of inflammation in the trachea and the direct relaxation of bronchial smooth muscle, but mechanisms regarding the antitussive effect of 1-MID are needed to fully elucidate.

### 4.11. Anticancer Activities

Plenty of reports have suggested that plant-derived agents have anticancer activity, such as *Catharanthus roseus*, *Taxus brevifolia*, *Podophyllum peltatum*, and *Robinia pseudoacacia* [145, 146]. To this end, as an animal-based traditional medicine, protein hydrolysate from OR (PHOR) showed favorable antiglioma activity. In human glioma C_6_ cell, 500 *μ*g/mL of PHOR significantly inhibited cell proliferation and colony formation as well as promoted apoptosis via downregulating Bcl-2 coupled with upregulating Bax and cleaved caspase-3. Moreover, intragastric administration of PHOR in a dose of 1.5 g/kg BW to a nude mouse model of glioma remarkably slowed glioma growth and increased the levels of TNF-*α*, IL-1*β*, and IL-6 through the activation of PI3K/AKT signaling [127].

In summary, globally, cancer is the second leading cause of morbidity and mortality, next to cardiovascular disease [147]. It is therefore important to grope for novel anticancer drugs. To the best of our knowledge, only one paper reported the antiglioma activity of PHOR, but it can provide a new clue for further studies and also inspire us that animal-based traditional medicine is an equally important source of anticancer drug discovery.

## 5. Safety Properties of *Oviductus ranae*

OR, a traditional Chinese medicine, has been consumed for hundreds of years in China. Although it has been listed as a drug in Chinese Pharmacopoeia since 1985, investigation of its safety evaluations is still lacking, and only few reports were published, which are listed in Table 3.

In our previous work, the safety properties of OR were evaluated both in mice and in rats by a series of tests including acute toxicity, subacute toxicity, Ames test, micronucleus test, and sperm malformation assay. The acute toxicity was investigated using intragastric administration of OR to mice in doses of 2.5, 5, 10, and 20 g/kg BW. The upper dose was 200 times higher than the human dose in clinical use and no abnormality and mortality were found within an observation period of 14 days. The subacute toxicity was conducted using intragastric administration of OR to rats in doses of 1.75, 3.5, and 7 g/kg BW for 28 days. It was noted that oral administration of OR had no effect on food intake, hematological parameters, biochemical markers, relative organ weights, and the microphotographs of organs and tissues. The Ames test was detected using four *Salmonella typhimurium* strains TA97, TA98, TA100, and TA102 at concentrations of 8, 40, 200, 1000, and 5000 *μ*g/plate. Results showed that no increase in number of revertants was found even at a concentration of 5000 *μ*g/plate in any tested bacterial strains with or without S-9 mix. The micronucleus test was evaluated using intragastric administration of OR to mice in doses of 2.5, 5, and 10 g/kg BW for 2 days. It was noted that no significant differences in MR (micronucleus rate) and PCE (polychromatic erythrocytes)/RBC (red blood cells) ratio between the control and OR-treated groups were observed. The sperm malformation assay was conducted using intragastric administration of OR to male mice in doses of 2.5, 5, and 10 g/kg BW for 5 days. Results showed that no statistical differences in sperm malformation rate were found in the OR-treated groups compared with control [19]. Then, the protein-rich extract from OR (PEOR) was prepared and analyzed; before bioactive evaluations, the acute toxicity of PEOR was conducted in doses of 5, 10, and 20 g/kg BW to preliminarily assess its edible safety. Results demonstrated that the maximal tolerated dose (MTD) of PEOR in mice was higher than 20 g/kg BW and oral administration of PEOR in a single dose of 20 g/kg BW had no effects on body weight, relative organ weight, biochemical parameters, and histopathological changes [84]. In the near future, the toxicity evaluations regarding multiple doses of PEOR will be performed to further verify its safety profiles.

Furthermore, Liu et al. assessed the acute toxicity and genotoxicity of compound Danggui-Shuangshen-Hamayou tablet (DSHT). It was observed that the MTD of DSHT in mice was higher than 20 g/kg BW and no genotoxicity was found during the period of experiment [148]. Cui et al. evaluated the genotoxicity of Hamayou soft capsule and found that this kind of functional food exhibited acceptable safety properties [149].

In a different experiment, Wang et al. investigated the heavy metals in the oviducts derived from *R. dybowskii* (OR), *R. chensinensis*, and *R. amurensis*, respectively. The analysis showed that there were significant differences in residual quantities of five heavy metals including stannum (Sn), cadmium (Cd), chromium (Cr), cuprum (Cu), and plumbum (Pb) in different frogs and the residual quantities of Cr and Cd were much higher than those of the three other heavy metals [150], indicating that it is also important to monitor the growing environment of *R. dybowskii*, thereby controlling the residual quantities of heavy metals under the maximal residue limit and guaranteeing the edible safety of OR.

In short, clinical applications and known investigations have confirmed the edible safety of OR, which provided a basic precondition of OR to be a source of nutraceuticals and functional foods.

## 6. *Oviductus ranae*-Based Nutraceutical Products in China

In China, OR enjoys a high reputation and is commonly sold in the form of dried raw materials, which can be eaten as foods directly after being macerated or stewed in water [25]. Unlike other traditional Chinese medicinal materials listed in Chinese Pharmacopoeia, OR is seldom consumed as a therapeutic drug but is usually served as a raw material for functional foods.  Table 4 exemplifies the current functional foods based on OR on the market approved by the China Food and Drug Administration (CFDA). It shows that approximately 90% of the market-available functional foods based on OR are used for immune enhancement and/or antifatigue, which are consistent with its ethnopharmacology and traditional uses which signify that OR can nourish the kidney, strengthen the essence, and moisten the lung to nourish Yin [24], due to the fact that tonic agents are customarily prescribed for the applications of immune enhancement and antifatigue [151, 152].

Some investigational nutraceutical products based on OR have concentrated on other functions including antioxidation, radiation protection, antiosteoporosis, alleviation of climacteric syndrome, and improvement of sleep. To enhance functions, OR was used together with other traditional Chinese medicines and/or nutraceuticals, such as ginseng, *Curcuma zedoaria*, *Rhizoma Dioscoreae*, *Lycium chinense*, *Angelica sinensis*, turtleback, *Astragalus mongholicus*, *Rhodiola rosea*, *Epimedii Folium*, *Radix Puerariae*, *Pleurotus citrinopileatus*, polysaccharides from *Acanthopanax senticosus*, ginsenoside, bilberry anthocyanins, chlorogenic acid, vitamin C, vitamin E, *Fritillaria ussuriensis*, *Semen Ziziphi spinosae*, *Polygala tenuifolia*, glycerine, florence oil, shea butter, and *Ophiopogon japonicus*. In addition, even lotion based on OR has also been prepared and evaluated for moisturizing and sunscreening (Table 5).

All in all, OR has been well recognized for its beneficial biological functions, especially immune enhancement and antifatigue, and OR-based functional foods have been fully developed compared with other tonic traditional Chinese medicines in China. In further development and expansion of OR-based nutraceuticals, some personally designed products should be considered. Personalized nutrition focuses on the relationship between genetic variants and diet to ameliorate physical status and/or prevent/cure diseases [164]. OR and its active principle-derived nutraceutical products should be diversified in their functions to cater to different needs. For example, liposoluble constituents from OR are rich in vitamin E and estrogens, which can be developed to antagonize aging or antagonize osteoporosis in female individuals with climacteric syndrome. However, water-soluble constituents from OR are mainly composed of proteins, which can be developed to antagonize oxidative stress or enhance immunity or antagonize fatigue in male and female individuals.

## 7. Conclusion

*Oviductus ranae* (OR) has been wildly used as a tonic in traditional Chinese medicine (TCM) for hundreds of years. Traditional uses of OR mainly include relieving some ailments such as debilitation, phthisis, cough, hematemesis, and night sweat. Although OR has been recorded in Chinese Pharmacopoeia as a traditional Chinese medicinal material since the year 1985, it is seldom prescribed as a medicine to cure diseases but usually consumed as nutraceuticals to optimize health.

The results of recent bioactive studies of OR have validated its traditional uses. Both *in vitro* and *in vivo* bioactive studies have indicated that OR exerts immunomodulatory, antioxidant, antifatigue, antiaging, estrogen-like, hepatoprotective, hypolipidemic, antiosteoporotic, antidepressant, antitumor, antitussive, expectorant, anti-inflammatory, and antiasthmatic activities. It is worthwhile to note that bioactive studies and clinical practices provide strong evidence for the functions of OR on immunomodulation, antioxidation, and antifatigue. In previous work, we also observed that protein-rich extracts from OR elicit significant antioxidant and antifatigue activities [84].

OR contains a large number of bioactive constituents including proteins, amino acids, polypeptides, steroids, fatty acids, phospholipids, nucleosides, vitamins, hydantoins, and mineral elements. Among these bioactive ingredients identified in OR, proteins, amino acids, polypeptides, and steroids are assumed to be the main bioactive components that attributed to the majority of biological functions of OR. However, most of the bioactive studies were carried out using OR or uncharacterized crude extracts from it. Therefore, the bioactivity-guided identification is still needed to fully characterize the bioactive compounds.

Based on clinical applications as well as our previous work [19], it is concluded that there is no noticeable toxicities of OR consumption. But as for the premature ovarian failure caused by high-dose consumption of OR in normal female mice [110], other studies are needed to affirm whether long-term and high-dose consumption of OR increases the incidence of ovarian cancer in normal individuals.

Due to beneficial functions and favorable safety properties, OR-based nutraceuticals and functional foods have been well developed in China, and approximately twenty OR-based functional foods have been approved on the market by the CFDA. Several OR-based nutraceutical products with multiple functions and purposes are being investigated, and personalized nutrition is the trend for further development of OR-based products.

In summary, the information reviewed here may provide evidence for the further uses of OR and its active principles as nutraceuticals and functional foods.

## Figures and Tables

**Table 1:** Bioactive constituents present in *Oviductus ranae*.

Class	Chemical ingredients	References
Mineral elements	K, Na, Ca, Mg, Fe, Mn, Zn, Cu, Sr, Cr, Mo, Se, P, Al, Ba, Ni, Co, V, Ag, Pb, and Cd	[26–29]
Amino acids	Asp, Thr, Ser, Glu, Pro, Gly, Ala, Cys, Val, Met, Ile, Leu, Tyr, Phe, His, Lys, Arg, and Trp	[30–32]
Proteins	170, 157, 128, and 49 kDa proteins	[33]
	116 kDa protein	[34, 35]
	66 kDa protein	[36]
	53~66 kDa polypeptides or proteins	[37]
	20.2 kDa protein	[38]
Steroids	Cholesterol	[39–42]
	7-Dehydrocholesterol	[41]
	Cholest-4-ene-3-one	[40, 42]
	Cholest-5-ene-3*β*, 7*β*-diol	[11, 43]
	3*β*-Hydroxyl-cholest-5-ene-7-one	[11, 40, 43]
	Cholesteryl palmitate	[44, 45]
	Cholestan-3,6-dione	[42]
	Stigmasterol	[41]
	Estradiol	[46]
	Progesterone	[46]
	Testosterone	[46, 47]
	Androsterone acetate	[46]
	Medrogestone	[46]
Fatty acids	Heptanoic acid	[48]
	7-Nonenoic acid	[48]
	10-Undecenoic acid	[48]
	Lauric acid	[48]
	Ficocerylic acid	[49]
	Myristic acid	[50]
	Pentadecanoic acid	[50]
	Palmitic acid	[50]
	Palmitoleic acid	[50]
	Hexadecadienoic acid	[49]
	14-Methyl-pentadecanoic acid	[48]
	Heptadecanoic acid	[50]
	Stearic acid	[50]
	Oleic acid	[50, 51]
	Linoleic acid	[50, 51]
	*α*-Linolenic acid	[51]
	11-Octadecenoic acid	[48]
	16-Methyl-heptadecanoic acid	[48]
	Arachidic acid	[50]
	Eicosadienoic acid	[49]
	Eicosatrienoic acid	[49]
	Arachidonic acid	[50]
	*cis*-5, 8, 11, 14, 17-Eicosapentaenoic acid	[50]
	Behenic acid	[50]
Phospholipids	Sphingomyelin	[52]
	Cephalin	[52]
	Phosphatidylcholine	[52]
Vitamins	Vitamin E	[53, 54]
	Vitamin A	[53]
	Vitamin D	[53]
	Vitamin B_1_	[53]
	Vitamin B_2_	[53]
	Vitamin C	[53]
	Vitamin K	[52]
	*β*-Carotene	[52]
Nucleosides	Inosine	[55]
Hydantoins	1-Methylhydantoin	[24, 56, 57]
Others	Polysaccharides	[58, 59]
	Human chorionic gonadotropin	[53, 54]
	Thyroxin	[53, 54]
	Triiodothyronine	[53]
	Parathyroid hormone	[54]

Abbreviations: K: potassium; Na: sodium; Ca: calcium; Mg: magnesium; Fe: ferrum; Mn: manganese; Zn: zinc; Cu: copper; Sr: strontium; Cr: chromium; Mo: molybdenum; Se: selenium; P: phosphorus; Al: aluminum; Ba: barium; Ni: nickel; Co: cobalt; V: vanadium; Ag: silver; Pb: lead; Cd: cadmium; Asp: aspartic acid; Thr: threonine; Ser: serine; Glu: glutamic acid; Pro: proline; Gly: glycine; Ala: alanine; Cys: cysteine; Val: valine; Met: methionine; Ile: isoleucine; Leu: leucine; Tyr: tyrosine; Phe: phenylalanine; His: histidine; Lys: lysine; Arg: arginine; Trp: tryptophan.

**Table 2:** Biological functions of *Oviductus ranae* and its active principles.

Healthy functions	Active principle/preparation	Model	Dosage	Results	Reference
Immunomodulatory activities	*Oviductus ranae*	Mice (*in vivo*)	0.67 and 1.33 g/kg BW, i.g., for 30 days	Enhance lymphocyte proliferation, antibody-producing cells, delayed-type hypersensitivity response, NK cell activity, and phagocytosis of mononuclear macrophages	[73]
	*Oviductus ranae* protein hydrolysate prepared by neutral protease	Splenic lymphocyte and RAW 264.7 cells (*in vitro*)	500 *μ*g/mL	Increase spleen lymphocyte proliferation, IL-2 production, macrophage phagocytosis, and NO production	[74]
	*Oviductus ranae* protein hydrolysates prepared by different proteases	RAW 264.7 cells (*in vitro*)	500 *μ*g/mL	Improve macrophage phagocytosis and NO production	[75]
	*Oviductus ranae* protein hydrolysate prepared by trypsin	Splenic lymphocyte and RAW 264.7 cells (*in vitro*)	50-800 *μ*g/mL	Increase spleen lymphocyte proliferation, macrophage phagocytosis, and NO production	[76]
	*Oviductus ranae* protein hydrolysates prepared by different proteases	RAW 264.7 cells (*in vitro*)	10-2000 *μ*g/mL	Enhance macrophage phagocytosis and productions of IL-1*β*, IL-6, TNF-*α*, NF-*κ*B, and NO; upregulate the mRNA and protein expression of iNOS	[77]
	Peptide from *Oviductus ranae*	Splenic lymphocyte (*in vitro*) Mice (*in vivo*)	20-100 mg/mL (*in vitro*); 600 mg/kg BW, i.g., for 30 days (*in vivo*)	Increase spleen lymphocyte proliferation and immune organ index	[78]
	Compound Renshen-Hamayou capsule	Mice (*in vivo*)	1.5 g/kg BW, i.g., for 45 days	Enhance delayed-type hypersensitivity response and spleen lymphocyte proliferation	[79]
	Hamayou soft capsule	Healthy male athletes aged from 18 to 24 years (*in vivo*)	Three times a day, 4 g each time, P.O., for 28 days	Increase CD_3_^+^, CD_4_^+^, and CD_4_^+^/CD_8_^+^ ratio	[80]
Antioxidant activities	*Oviductus ranae*	Mice exposed to cold stress (*in vivo*)	0.5 and 2.5 g/kg BW, i.g., for 28 days	Increase SOD; decrease MDA	[81]
	*Oviductus ranae*	Young and aging mice (*in vivo*)	0.45, 1.35, and 2.25 g/kg BW, i.g., for 15, 30, and 45 days, respectively	Upregulate the expression of Cu/Zn-SOD and GPx4 genes	[82]
	Serum from *Oviductus ranae-*treated rats	H_2_O_2_-induced oxidative stress in rat ovarian granulosa cells (*in vitro*)	0.09, 0.27, and 0.81 g/kg BW, i.g., for 7 days	Decrease apoptosis by reducing ROS production and improving mitochondrial membrane potential through downregulating p53, Bax, caspase-3, and caspase-9 and upregulating Bcl-2; weaken phosphorylation of JNK and p38 MAPK; enhance ERK1/2 phosphorylation	[83]
	Water-soluble proteins from *Oviductus ranae*	Hydroxyl, DPPH, and superoxide anion radicals and reducing power (*in vitro*)	1-5 mg/mL; 2-10 mg/mL; 8-16 mg/mL	Weak radical-scavenging capacities towards hydroxyl, DPPH, and superoxide anion as well as reducing power to ferric iron	[84]
		Ethanol-induced oxidative stress in mice (*in vivo*)	0.1, 0.2, and 0.4 g/kg BW, i.g., for 30 days	Increase T-SOD and GSH; decrease MDA and PCO	
	Four proteins from *Oviductus ranae*	DPPH and hydroxyl radicals and phosphatidylcholine liposome (*in vitro*)	0.01-10 mg/mL	Water-soluble and salt-soluble proteins possess stronger *in vitro* antioxidant capacities than gliadin and alkali-soluble protein.	[85]
	Two polypeptides from *Oviductus ranae* protein	DPPH, hydroxyl, and superoxide anion radicals (*in vitro*)	2-12 mg/mL	Polypeptide with molecular weight of 1~5 kDa exerts stronger *in vitro* antioxidant activity than the polypeptide with molecular weight of 5~8 kDa.	[86]
	*Oviductus ranae*/water-soluble proteins and water-insoluble constituents from *Oviductus ranae/*three hydrolysates from *Oviductus ranae* water-soluble protein	DPPH, hydroxyl, and superoxide anion radicals (*in vitro*)	2-10 mg/mL	Water-soluble proteins display stronger *in vitro* antioxidant capacity than *Oviductus ranae* and water-insoluble constituents. After hydrolysis, the activity was increased and protein hydrolysates prepared by neutral protease show the highest activity.	[87]
	Compound Renshen-Hamayou capsule	Aging rats (*in vivo*)	1.25 g/kg BW, i.g., for 60 days	Decrease MDA; increase GSH-Px activity	[88]
	Compound Linwayou granule	X-ray-induced oxidative stress in mice (*in vivo*)	100 and 200 mg/kg BW, i.g., for 7 days	Increase SOD and GSH-Px activities	[89]
Antifatigue activities	*Oviductus ranae*	Mice (*in vivo*)	0.25, 0.5, and 1.0 g/kg BW, i.g., for 30 days	Prolong the endurance time of FST, increase liver glycogen, and decrease BLA	[90]
	*Oviductus ranae* and its extracts	Hydrocortisone-induced kidney Yang deficiency syndrome in mice (*in vivo*)	OR: 680 mg/kg BW; PEEOR: 136 mg/kg BW for 7 days	OR and PEEOR can increase body temperature and body weight and prolong the endurance time of rotarod test, hypoxia tolerance test, and FST.	[91]
	*Oviductus ranae*	High-intensity exercise training rats (*in vivo*)	0.5, 1.0, and 3.0 g/kg BW, i.g., for 6 weeks	Increase body weight, endurance time of FST, testosterone, hemoglobin, and hepatic and muscle glycogen; decrease BUN	[92]
	Water-soluble proteins from *Oviductus ranae*	Mice (*in vivo*)	0.075, 0.15, and 0.3 g/kg BW, i.g., for 30 days	Increase hepatic glycogen; decrease BLA	[93]
	Protein hydrolysates from *Oviductus ranae*	Mice (*in vivo*)	0.052, 0.52, and 1.56 g/kg BW, i.g., for 30 days	Increase the endurance time of FST; decrease BUN and BLA	[94]
	Peptides from *Oviductus ranae*	Mice (*in vivo*)	100, 200, and 400 mg/kg BW, i.g., for 30 days	Prolong the endurance time of FST, rotarod test, and pole-jumping test; reduce BUN and BLA	[95]
	Water-insoluble components from *Oviductus ranae*	Mice (*in vivo*)	0.25, 0.5, and 1.0 g/kg BW, i.g., for 30 days	Increase the endurance time of FST and hepatic glycogen; decrease BLA	[96]
	Linwayou soft capsule	Mice (*in vivo*)	1.0, 2.0, and 3.0 g/kg BW, i.g., for 30 days	Increase the endurance time of FST and hepatic glycogen; decrease BLA and BUN	[97, 98]
	Compound Linwayou-Yuhuangmo capsule	Mice (*in vivo*)	0.7, 1.4, and 2.1 g/kg BW, i.g., for 30 days	Increase the endurance time of FST and hepatic glycogen; decrease BLA and BUN	[99]
Antiaging activities	*Oviductus ranae*	*Drosophila melanogaster* (*in vivo*)	0.34 and 0.68 mg/g culture medium	Increase life-span, resistance to cold, and lipofuscin	[100]
	*Oviductus ranae*	*D*-Galactose-induced aging mice (*in vivo*)	0.1, 0.2, and 0.4 g/kg BW, i.g., for 43 days	Decrease MDA and XOD; increase MPO and NO	[101]
	*Oviductus ranae*	*D*-Galactose-induced aging rats (*in vivo*)	0.45, 0.9, and 1.8 g/kg BW, i.g., for 28 days	Upregulate the expressions of cyclin D1, CDK6, and p15; downregulate p16 and p21	[102–105]
	Water-soluble proteins from *Oviductus ranae*	*D*-Galactose-induced aging mice (*in vivo*)	0.075, 0.15, and 0.30 g/kg BW, i.g., for 30 days	Decrease MDA; increase SOD	[106]
	Water-insoluble constituents from *Oviductus ranae* and their hydrolysates	*D*-Galactose-induced aging mice (*in vivo*)	0.15 and 0.3 g/kg BW, i.g., for 30 days	Water-insoluble constituents from *Oviductus ranae* can reduce MDA. Hydrolysates can decrease MDA and increase SOD and GSH-Px.	[107]
	Hamayou capsule	*D*-Galactose-induced aging mice (*in vivo*)	1.4 and 2.8 g/kg BW, i.g., for 28 days	Alleviate pathological changes of ovary and uterus; increase SOD, organ indexes of ovarian and uterine, estradiol and estrous cycle; decrease MDA	[108]
Estrogen-like activities	Serum from *Oviductus ranae*-treated rats	Rat ovarian granulosa cells (*in vitro*)	4.5 g/kg BW, i.g., for 5 days	Increase proliferation and E2 secretion, inhibit apoptosis, and protect ovarian granulosa cells	[109]
	*Oviductus ranae*	Mice (*in vivo*)	0.05 and 0.2 g/kg BW, i.g., for 37 days	Increase E2, FSH, T, and P; increase the thickness of uterine wall and the number of corpora atretica; enhance the expression of FSHR gene	[110]
	Compound *Rhizoma curcumae*-*Oviductus ranae*	Ovariectomized rats (*in vivo*)	0.5, 1.0, and 2.0 g/kg BW, i.g., for 12 weeks	Increase E2, T, P, and IL-2; decrease FSH and LH; enhance the expression of ER	[111]
Hepatoprotective activities	*Oviductus ranae*	CCl_4_-induced liver fibrotic rats (*in vivo*)	1.5 and 3.0 g/kg BW, i.g., for 8 weeks	Decrease ALT, AST, ALP, GGT, Hyp, and MDA; increase SOD and GSH-Px	[112]
	*Oviductus ranae*	Ethanol-induced liver fibrotic rats (*in vivo*)	3 g/kg BW, i.g., for 8 weeks	Increase glycogen granule; hepatocyte nuclear appeared to be large and round.	[113]
	*Oviductus ranae*	High-intensity exercise training mice (*in vivo*)	0.9, 4.5, and 9.0 g/kg BW, i.g., for 28 days	Decrease MDA in the liver, increase SOD in the liver, and improve the ultrastructure of impaired hepatic cells	[114]
Hypolipidemic activities	*Oviductus ranae*	High-fat-diet-induced hyperlipidemic rats (*in vivo*)	0.05, 0.1, and 0.2 g/kg BW, i.g., for 30 days	Decrease TC and TG	[115]
	Water-insoluble constituents and their hydrolysates from *Oviductus ranae*	High-fat-diet-induced hyperlipidemic rats (*in vivo*)	0.05, 0.1, and 0.2 g/kg BW, i.g., for 30 days	Decrease TC and TG; the effects of hydrolysates were superior to water-insoluble constituents.	[116, 117]
	Water-soluble proteins from *Oviductus ranae*	High-fat-diet-induced fatty rats (*in vivo*)	0.05, 0.1, and 0.2 g/kg BW, i.g., for 30 days	Decrease TC and TG	[118]
Antiosteoporotic activities	*Oviductus ranae*	Ovariectomized rats (*in vivo*)	0.05 g/kg BW, i.g., for 12 weeks	Increase calcium, estrogen, BMD, bone scan index, femoral weight, W.th, and osteocalcin; decrease ALP, phosphorus, and BGP; enhance the proliferation of osteoblastic cells and the formation of mineralized nodes; reduce the number of TRAP-positive cells	[119]
	*Oviductus ranae*	Women with postmenopausal osteoporosis (*in vivo*)	1 g per day, P.O., for 6 months	Improve clinical signs and symptoms; increase E2; decrease BALP, IL-6, and BGP	[120]
Antidepressant activities	Petroleum ether extract of *Oviductus ranae*	Behavioral despair model of depression in mice (*in vivo*) Antagonism of reserpine-induced hypothermia in mice (*in vivo*)	100, 300, and 900 mg/kg BW, i.g., for 8 days	Decrease the dead time of FST and TST, CORT, and akinesia; increase body temperature	[121]
	Petroleum ether extract of *Oviductus ranae*	Chronic mild stress model of depression in rats (*in vivo*)	30, 100, and 300 mg/kg BW, i.g., for 21 days	Increase body weight, motion distance of OFT, and sucrose preference; decrease CORT; upregulate the expression of BDNF protein	[122]
	1-Methylhydantoin	Chronic forced swim stress-induced depression in rats (*in vivo*)	20, 40, and 80 mg/kg BW, i.g., for 14 days	Increase body weight and sucrose preference; decrease CORT; upregulate the expression of BDNF protein	[123]
Antitussive, expectorant, antiasthmatic, and anti-inflammatory activities	*Oviductus ranae* and its different extracts	Sulfur dioxide and ammonia-induced cough in mice (*in vivo*)	OR: 680 mg/kg BW; PEEOR: 68 and 136 mg/kg BW; MEOR: 85 and 170 mg/kg BW, i.g., for 3 and 7 days, respectively	Prolong the incubation period of cough and reduce cough frequency (efficiency: OR > MEOR > PEEOR); increase phenol red expectoration (efficiency: OR > PEEOR > MEOR) and sputum ejection (efficiency: PEEOR > OR > MEOR)	[124]
	*Oviductus ranae*	Ovalbumin-induced allergic asthma in mice (*in vivo*)	0.05 and 0.5 g/kg BW, i.g., for 8 weeks	Decrease inflammatory cell count, IL-4, IL-5, and IFN-*γ* in BALF	[125]
	1-Methylhydantoin	Ovalbumin-induced allergic asthma in rats (*in vivo*)	40 and 80 mg/kg BW, i.g., for 7 days	Decrease IL-5, eotaxin, and eosinophil count in BALF	[126]
		Acetylcholine-induced bronchial asthma in guinea pigs (*in vivo*)	30 and 60 mg/kg BW, i.g., for 3 days	Prolong the incubation period of asthma	
		Histamine-induced contraction of guinea pig tracheal smooth muscle (*in vitro*)	0.5 and 1.0 g/L	Increase antispasmodic percentage	
		Ammonia-induced cough in mice (*in vivo*)	50 and 100 mg/kg BW, i.g., for 3 days	Prolong the incubation period of cough and reduce cough frequency	
		Citric acid-induced cough in guinea pigs (*in vivo*)	30 and 60 mg/g BW, i.g., for 3 days	Prolong the incubation period of cough and reduce cough frequency	
Anticancer activities	Protein hydrolysates from *Oviductus ranae*	Human glioma C_6_ cell (*in vitro*)	500 *μ*g/mL	Inhibit glioma cell proliferation and colony formation; promote apoptosis in glioma cell; downregulate Bcl-2; upregulate Bax and cleaved caspase-3	[127]
		Glioma model in nude mice (*in vivo*)	1.5 g/kg BW, i.g.	Inhibit glioma growth; increase IL-1*β*, IL-6, and TNF-*α*; upregulate p-PI3K, AKT, and p-AKT	

Abbreviations: BW: body weight; i.g.: intragastric administration; CD: cluster of differentiation; IL-2: interleukin-2; NK: natural killer; NO: nitric oxide; IL-1*β*: interleukin-1*β*; IL-6: interleukin-6; TNF-*α*: tumor necrosis factor-*α*; NF-*κ*B: nuclear factor-*κ*B; mRNA: messenger ribonucleic acid; iNOS: inducible nitric oxide synthase; P.O.: oral administration; T-SOD: total superoxide dismutase; SOD: superoxide dismutase; MDA: malondialdehyde; PCO; GPx4: glutathione peroxidase 4; ROS: reactive oxygen species; MAPK: mitogen-activated protein kinases; DPPH: 1,1-diphenyl-2-picrylhydrazyl; GSH: glutathione; GSH-Px: glutathione peroxidase; FST: forced swimming test; BUN: blood urea nitrogen; BLA: blood lactic acid; H_2_O_2_: hydrogen peroxide; XOD: xanthine oxidase; MPO: myeloperoxidase; CDK-6: cyclin-dependent kinases-6; E2: estradiol; FSH: follicle-stimulating hormone; T: testosterone; P: progesterone; FSHR: follicle-stimulating hormone receptor; T*β*RI: type I transform growth factor *β* receptor; T*β*RII: type II transform growth factor *β* receptor; LH: luteinizing hormone; ER: estrogen receptor; ALT: alanine aminotransferase; AST: glutamic-oxalacetic transaminase; ALP: alkaline phosphatase; GGT, *γ*-glutamyltransferase; Hyp: hydroxyproline; CCl_4_: carbon tetrachloride; TC: total cholesterol; TG: triglyceride; BGP: bone gla protein; BMD: bone mineral densities; W.th: wall thickness; TRAP: tartrate-resistant acid phosphatase; BALP: bone alkaline phosphatase; IL-6: interleukin-6; Runx2: runt-related transcription factor 2; BMP2: bone morphogenetic protein 2; TST: tail suspension test; CORT: corticosterone; OFT: open field test; BDNF: brain-derived neurotrophic factor; PEEOR: petroleum ether extract from *Oviductus ranae*; MEOR: methanol extract from *Oviductus ranae*; IL-4: interleukin-4; IL-5: interleukin-5; IFN-*γ*: interferon-*γ*; BALF: bronchial alveolus lavage fluid; IL-1*β*: interleukin-1*β*.

**Table 3:** Safety properties of *Oviductus ranae* and its active principle as well as preparations.

Active principle/preparation	Main constituents	Tests	Model	Dosage	Results	Reference
Raw material	*Oviductus ranae*	Acute toxicity	Mice (*in vivo*)	2.5-20 g/kg BW, i.g., single-dose administration, followed by an observation of 14 days	MTD > 20 g/kg BW	[19]
		Subacute toxicity	Rats (*in vivo*)	1.75, 3.5, and 7 g/kg BW, i.g., for 28 days	NOAEL > 7 g/kg BW	
		Ames test	*Salmonella typhimurium* strains (*in vitro*)	8-5000 *μ*g/plate	None	
		Micronucleus test	Mice (*in vivo*)	2.5, 5, and 10 g/kg BW, i.g., for 2 days	None	
		Sperm malformation assay	Mice (*in vivo*)	2.5, 5, and 10 g/kg BW, i.g., for 5 days, followed by an observation of 30 days	None	
Active principle	Protein-rich extract from *Oviductus ranae*	Acute toxicity	Mice (*in vivo*)	5, 10, and 20 g/kg BW, i.g., single-dose administration, followed by an observation of 14 days	MTD > 20 g/kg BW	[84]
Compound Danggui-Shuangshen-Hamayou tablet	Ginseng, *Angelica sinensis*, *Salviae miltiorrhizae*, and *Oviductus ranae*	Acute toxicity	Mice (*in vivo*)	20 g/kg BW, i.g., single-dose administration, followed by an observation of 7 days	MTD > 20 g/kg BW	[148]
		Micronucleus test	Mice (*in vivo*)	2.5, 5, and 10 g/kg BW, i.g., for 2 days	None	
		Sperm malformation assay	Mice (*in vivo*)	2.5, 5, and 10 g/kg BW, i.g., for 5 days, followed by an observation of 30 days	None	
Hamayou soft capsule	*Oviductus ranae*	Ames test	*Salmonella typhimurium* strains (*in vitro*)	0.313-5 g/plate	None	[149]
		Micronucleus test	Mice (*in vivo*)	0.94-7.5 g/kg BW, i.g., for 4 days	None	
		Sperm malformation assay	Mice (*in vivo*)	1.88, 3.75, and 7.5 g/kg BW, i.g., for 5 days, followed by an observation of 30 days	None	

Abbreviations: BW: body weight; i.g.: intragastric administration; MTD: maximal tolerated dose; NOAEL: no-observed-adverse-effect levels.

**Table 4:** Current functional foods based on *Oviductus ranae* on the market approved by the China Food and Drug Administration.

Brand ®	Dosage form	Main constituents	Health functions	Approval number
Tongrentang	Granule	*Oviductus ranae*	Antifatigue and immune enhancement	SHIJIANZI 2002-0060
Shiyuan	Granule	*Oviductus ranae* and ginseng extract	Antifatigue and immune enhancement	SHIJIANZI G20130137
Yishoutang	Soft capsule	*Oviductus ranae*, evening primrose oil, vitamin E, and tea polyphenols	Blood lipid regulation and antiaging	SHIJIANZI 2002-0193
Yicaotang	Liquid	*Oviductus ranae*	Antifatigue and immune enhancement	SHIJIANZI G20150785
Biyuantang	Soft capsule	*Oviductus ranae*	Antifatigue	SHIJIANZI G20100381
Xuejian	Soft capsule	*Oviductus ranae*	Antifatigue	SHIJIANZI 2002-0620
Jizhensanbao	Soft capsule	*Oviductus ranae* and vitamin E	Immune enhancement	SHIJIANZI G20130091
Wantong	Liquid	*Oviductus ranae*	Immune enhancement	SHIJIANZI 2003-0219
Xingjing	Capsule	*Oviductus ranae*	Immune enhancement	SHIJIANZI 2002-0024
Chunyuanwabao	Granule	*Oviductus ranae*	Immune enhancement and antifatigue	SHIJIANZI G20150654
Yisheng	Powder	*Oviductus ranae*	Immune enhancement	SHIJIANZI G20120426
Shengjixin	Granule	*Oviductus ranae*	Immune enhancement and antifatigue	SHIJIANZI G20150940
Yuanbo	Soft capsule	*Oviductus ranae* and vitamin E	Antifatigue	SHIJIANZI G20120244
Yuanbo	Soft capsule	*Oviductus ranae* and vitamin E	Immune enhancement	SHIJIANZI G20110405
Dongfangyaolin	Tablet	*Oviductus ranae*, ginseng, *Angelica sinensis*, the root of red-rooted salvia, and grape seed extract	Antichloasma	SHIJIANZI G20100592
Tangsong	Soft capsule	*Oviductus ranae* and collagen protein	Immune enhancement	SHIJIANZI G20140677
Hongming	Soft capsule	*Oviductus ranae*	Antifatigue	SHIJIANZI G20040428
Beijiang	Capsule	*Oviductus ranae*	Immune enhancement	SHIJIANZI 2003-0258
Yongli	Soft capsule	*Oviductus ranae*	Antifatigue	SHIJIANZI 2000-0219
Jizhu	Soft capsule	*Oviductus ranae* and American ginseng	Immune enhancement	SHIJIANZI G20041071

The information was obtained from http://samr.cfda.gov.cn/WS01/CL0001/; the search keywords were “*Oviductus ranae*”, “Hamayou”, and “Hashimayou”.

**Table 5:** Some investigational nutraceutical products based on *Oviductus ranae*.

Preparation name	Dosage form	Main constituents	Function	References
Compound Linwayou-Renshen capsule	Capsule	*Oviductus ranae* and ginseng	Antioxidation	[88]
Yifuning soft capsule	Soft capsule	*Oviductus ranae* and *Curcuma zedoaria*	Treat climacteric syndrome and postmenopausal osteoporosis	[153, 154]
Compound Liuwei Xueha tablet	Tablet	*Oviductus ranae*, ginseng, *Rhizoma Dioscoreae*, *Lycium chinense*, *Angelica sinensis*, and turtleback	Antiaging and antioxidant activities	[155]
Compound Renshen-Hamayou capsule	Capsule	*Oviductus ranae*, ginseng, *Astragalus mongholicus*, and *Rhodiola rosea*	Immune enhancement	
Shenlai tablet	Tablet	*Oviductus ranae*, *Rhodiola rosea*, *Astragalus mongholicus*, *Epimedii Folium*, *Radix Puerariae*, and *Lycium chinense*	Antifatigue	[156]
Compound Linwayou-Yuhuangmo capsule	Capsule	*Oviductus ranae* and *Pleurotus citrinopileatus*	Antifatigue	[99]
Compound Linwayou granule	Granule	*Oviductus ranae*, polysaccharides from *Acanthopanax senticosus*, ginsenoside, bilberry anthocyanins, chlorogenic acid, vitamin C, and vitamin E	Radiation protection	[89, 157–159]
Compound Linwayou-Pingbeimu soft capsule	Soft capsule	*Oviductus ranae* and *Fritillaria ussuriensis*	Immune enhancement	[160]
Compound Suanzaoren-Linwayou capsule	Capsule	*Oviductus ranae*, *Semen Ziziphi spinosae*, and *Polygala tenuifolia*	Improve sleep	[161]
Linwayou moisturizing emulsion	Emulsion	*Oviductus ranae*, glycerine, florence oil, and shea butter	Moisturizing and sunscreening	[162]
Compound Renshen-Linwayou soft capsule	Soft capsule	*Oviductus ranae*, ginseng, and *Ophiopogon japonicus*	Antifatigue	[163]
